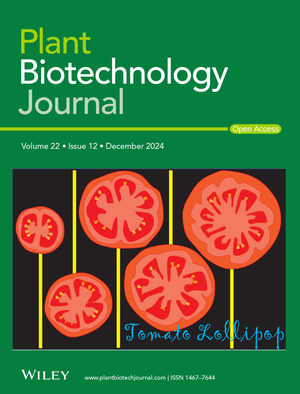# Issue Information

**DOI:** 10.1111/pbi.14090

**Published:** 2024-11-25

## Abstract

Front cover image:

Tomato Lollipop: Gene editing improves tomato to be as sweet as lollipops. Sugar accumulation in fruits gradually decreases during tomato domestication process of selection for large fruits. A natural variation of Lin5 in modern tomato cultivar has been substituted by the variation of its wild ancestor through prime editing, which results in a significant increase in fruit sugar accumulation. Cover illustration refers to the article published in this issue (Wang et al., pp. 3520–3522).